# High voltage pulsed current in collagen realignment, synthesis, and angiogenesis after Achilles tendon partial rupture

**DOI:** 10.1590/bjpt-rbf.2014.0167

**Published:** 2016-06-16

**Authors:** Érika P. Rampazo, Richard E. Liebano, Carlos Eduardo Pinfildi, Roberta A. C. Folha, Lydia M. Ferreira

**Affiliations:** 1 Departamento de Cirurgia Plástica, Universidade Federal de São Paulo (UNIFESP), São Paulo, SP, Brazil; 2 Departamento de Fisioterapia, Universidade Federal de São Carlos (UFSCar), São Carlos, SP, Brazil; 3 Departamento de Ciências do Movimento Humano, UNIFESP, Santos, SP, Brazil

**Keywords:** electrical stimulation, tendinopathy, healing, physical agents, tissue repair, physical therapy

## Abstract

**Objective:**

To verify the efficacy of high voltage pulsed current in collagen realignment and synthesis and in angiogenesis after the partial rupturing of the Achilles tendon in rats.

**Method:**

Forty male Wistar rats were randomized into four groups of 10 animals each: sham, cathodic stimulation, anodic stimulation, and alternating stimulation. Their Achilles tendons were submitted to direct trauma by a free-falling metal bar. Then, the treatment was administered for six consecutive days after the injury. In the simulation group, the electrodes were positioned on the animal, but the device remained off for 30 minutes. The other groups used a frequency of 120 pps, sensory threshold, and the corresponding polarity. On the seventh day, the tendons were removed and sent for histological slide preparation for birefringence and Picrosirius Red analysis and for blood vessel quantification.

**Results:**

No significant difference was observed among the groups regarding collagen realignment (types I or III collagen) or quantity of blood vessels.

**Conclusion:**

High voltage pulsed current for six consecutive days was not effective in collagen realignment, synthesis, or angiogenesis after the partial rupturing of the Achilles tendon in rats.

## BULLET POINTS

- HVPC did not improve collagen realignment of the Achilles tendon in rats.
- HVPC did not increase collagen synthesis of the Achilles tendon in rats.
- HVPC did not favor angiogenesis of the Achilles tendon in rats.
- It is premature to use the HVPC with the purpose of improving tendon healing.

## Introduction

Tendon lesions are characterized by an inflammatory condition in the paratendon, known as paratendinitis, or a collagen fiber degenerative process, known as tendinosis, in addition to total or partial rupture^1^. Intratendinous degenerative change is strongly associated with both chronic tendinopathies and spontaneous tendon rupture. Spontaneous tendon rupture, i.e., the sudden rupture of a tendon without preceding clinical symptoms, is a relatively common occurrence in sports, particularly in the recreational athlete, and tendon disorders constitute a major problem in sports and occupational medicine^2^.

The Achilles tendon is the largest and strongest tendon in the body and is the confluence of the gastrocnemius and the soleus muscles^1^. It plays an important role in the biomechanics of the lower extremity^1^ and it can withstand great forces^1^. Despite this, it is one of the most frequently injured tendons in the human body^3^. Much research has been performed to elucidate the etiology of a rupture of the Achilles tendon, but its true nature still remains unclear^4^.

After the injury, the healed tendon is functionally weaker when compared to the pre-injury state due to several factors related to the healing process, such as poor tissue vascularization. Hence, recurrent injuries or chronification risks are greater^5^.

The high incidence of these injuries, the fact that the healing process may not occur completely, and the long healing time justify the necessity for further studies to improve tendon healing and reduce the rehabilitation time for returning to regular activities^6^. We chose high voltage pulsed current (HVPC) because it is an important resource in tissue healing and there are few studies that have investigated its effects on tendon healing. To date, only Owoeye et al.^7^ have studied the effect of HVPC on tendon healing, and they observed that positive polarity increased the strength of the tendon against rupturing after a tenotomy in rats. Thus, the hypothesis of the study was that HVPC would favor tendon healing in the acute stage to increase collagen realignment and synthesis or angiogenesis.

Given the importance of this electrical current in tissue healing, the objective of the present study was to verify the efficacy of HVPC in collagen realignment and synthesis and in angiogenesis after a partial rupture of the Achilles tendon in rats.

## Method

### Animals

All of the animal experiments were approved by the Animal Care and Use Committee (protocol number 1613/10) of Universidade Federal de São Paulo (UNIFESP), São Paulo, SP, Brazil. All of the animals received humane care in strict compliance with the “Principles of laboratory animal care” (NIH publication No. 86-23, revised 1985).

The sample consisted of 40 adult male Wistar rats (Rattus norvegicus), aged eight and nine weeks, with a body mass of 260-320g. The rats were housed in individual polypropylene cages, on a 12:12h light-dark cycle and fed standard rat chow and water ad libitum. The temperature was controlled at 22 °C.

The 40 animals were randomly allocated into four groups of 10 rats each: sham group, cathodic stimulation group, anodic stimulation group, and alternating stimulation group. After the animals were submitted to the injury procedure, numbers from 1 to 40 were drawn by lottery to identify and allocate the animals into the respective groups previously determined by a computerized randomization system.

### Procedure to induce a partial rupture of the Achilles tendon

The animals were anesthetized with an intraperitoneal injection of ketamine hydrochloride (0.2 cm^3^) and xylazine hydrochloride (0.1 cm^3^). The hair over and around the right Achilles tendon and the dorsal region was removed. The animals were positioned on the injury device and a light traction was exerted on the calcaneal region with the ankle in dorsiflexion until the back of the paw touched the base of the device. A metal bar weighing 186g was released perpendicularly on the tendon of the animal from a height of 20 cm, corresponding to a potential energy of 364.9 mJ^8^ ^-^ ^11^.

### Application of high voltage pulsed current

The simulation or application of HVPC was performed two minutes after the injury, then at the same time and by the same researcher for six consecutive days. A Neurodyn High Volt® device with high-voltage pulsed, monophasic, twin-peak electrical current was used (Indústria Brasileira de Equipamentos Médicos, Ibramed®, Amparo, SP, Brazil).

The animals were positioned in ventral decubitus, and then silicon rubber electrodes with gel-covered surfaces (carboxyvinyl polymer) were placed to conduct the electrical stimulus (Carbogel®, São Paulo, SP, Brazil).

**Figure 1:**
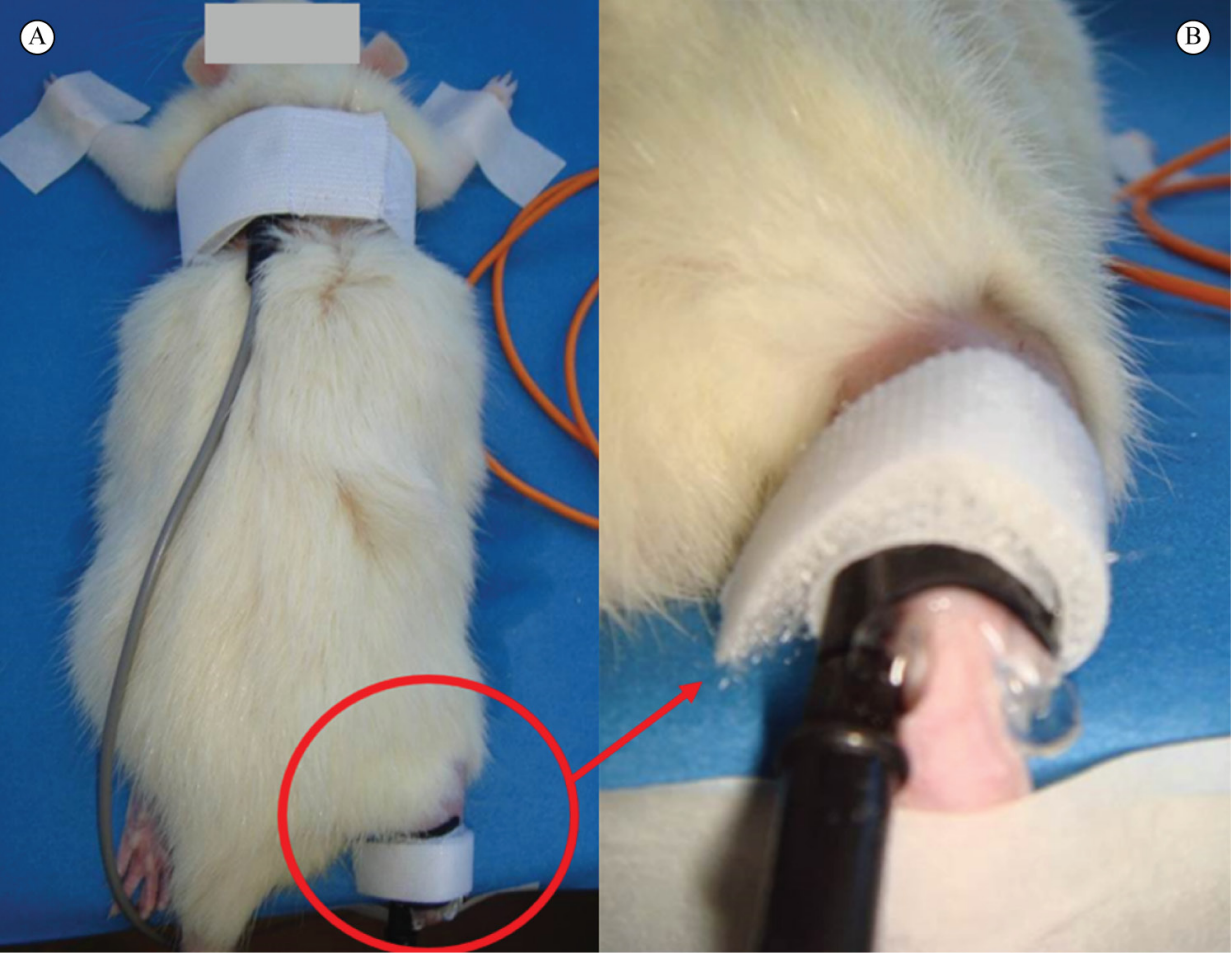
High voltage pulsed current application. (A) Superior view of dispersive electrode on the back and active electrode on the Achilles tendon at the time of the current application; (B) Amplified view of the active electrode on the Achilles tendon at the time of the current application.

The dispersive electrode (3.0 cm × 5.0 cm) was positioned on the back of the rat and held in place with an elastic band with Velcro straps at the ends. The right pelvic member was kept in dorsiflexion with adhesive tape on the plastic polystyrene block. The active electrode was positioned on the Achilles tendon and kept in place by Velcro straps (Figure 1).

In the sham group, the electrodes were positioned and kept in place for 30 minutes, but the device remained off. The following parameters were chosen in the HVPC application groups: 120 pps frequency, pulse duration of two twin pulses of 5 µs each with a 100 µs interval between them, 30 minutes of application at sensory threshold, and the polarity of the active electrode was selected according to the corresponding group. Negative polarity was selected in the cathodic stimulation group. Positive polarity was selected in the anodic stimulation group and negative polarity was selected in the alternating stimulation group on the first 3 days and then positive polarity on the following 3 days. The mean value of electrical charge at the electrode was 0.16 µC/impulse and 19.2 µC/second.

### Tendon excision and sample preparation

There was no loss of animals, and 6 days after a trauma-induced injury, ten animals per group were anesthetized to remove their Achilles tendons. After the Achilles tendons were removed, the animals were euthanized by overdose of anesthesia. Two incisions were made in each animal: one in the musculotendinous junction and another to the calcaneal insertion. After removal, the calcaneal tendon was rinsed in 0.9% saline solution and affixed to a cork surface by two pins. The first pin was placed on the base of the tendon (osteotendinous junction) and the second pin was placed on the gastrocnemius muscle. The Achilles tendons were placed in containers with 10% buffered formaldehyde for two hours. After this period, the tendons were removed from the cork surface and each sample was wrapped in filter paper and placed inside a small box, which was immersed in 10% buffered formaldehyde for 24 hours, and then they were dispatched to start the histological slide preparation. The sample was prepared according to the following criteria: 7 µm to the birefringence slides, 3 µm picrosirius slides and 5 µm to hematoxylin and eosin stained slides. The knives used were Leica 818 high profile microtome blades (Leica Microsystems GmbH, Ernst-Leitz, Straße, Wetzlar, Germany).

### Histological evaluations

The histological slides were selected randomly and classified with codes so as not to identify the group to which an animal was previously allocated.

#### Birefringence measurements

Birefringence optical retardation (OR) measurements were taken in monochromatic light (546 nm) using a light microscope (Leica Microsystems) with a POL 10x/0.22 eyepiece, a 0.9 condenser, and a compensator (k/4, Senarmont). Prior to the birefringence analysis, all of the histological slides were immersed in distilled water for 30 minutes. During the measurements, the longitudinal axis of the tendons was positioned at 45º to the microscope polarizer^12^. The resulting measurements, in degrees, were converted into nanometers (nm) by multiplying the values by 3.03^6^ ^,^ ^9^ ^,^ ^13^.

#### Picrosirius Red staining

The tissue stained with Picrosirius Red was examined by polarized light microscopy (Nikon E-800 microscope, London, UK) for the presence of thin type III collagen fibers (green) and thick type I collagen fibers (red and yellow). The images were captured with a CoolSNAP-Pro cf digital camera (Media Cybernetics Inc., Rockville, MD, USA) and were imported to the Image-Pro Plus 4.5 software (Media Cybernetics Inc.). The areas corresponding to each polarization were added by slides and the percentage of each type of polarization was calculated in relation to the total area analyzed. The results were expressed as the mean percentage of each type of collagen fiber.

#### Blood vessel quantification

The slides stained with hematoxylin and eosin were used to count blood vessels. The images were captured with the CoolSNAP-Pro cf camera (Media Cybernetics Inc.) attached to the Nikon E-800 microscope (100x magnification – 10x lens). The number of vessels in the tendons’ central area was then counted with the Image-Pro Plus 4.5 software. The total of vessels counted per slide was divided by the tendons’ central area, and the number of vessels per mm^2^ was obtained and then the average number of vessels/mm^2^ was calculated per group.

### Statistical analyses

The statistical analysis of data was performed using the BioEstat program, version 5.3. The normality of data distribution was verified using the Shapiro-Wilk test. Subsequently, one-way analysis of variance (ANOVA) was used for group comparisons. Statistical significance was set at p<0.05 for all analyses.

## Results

**Figure 2:**
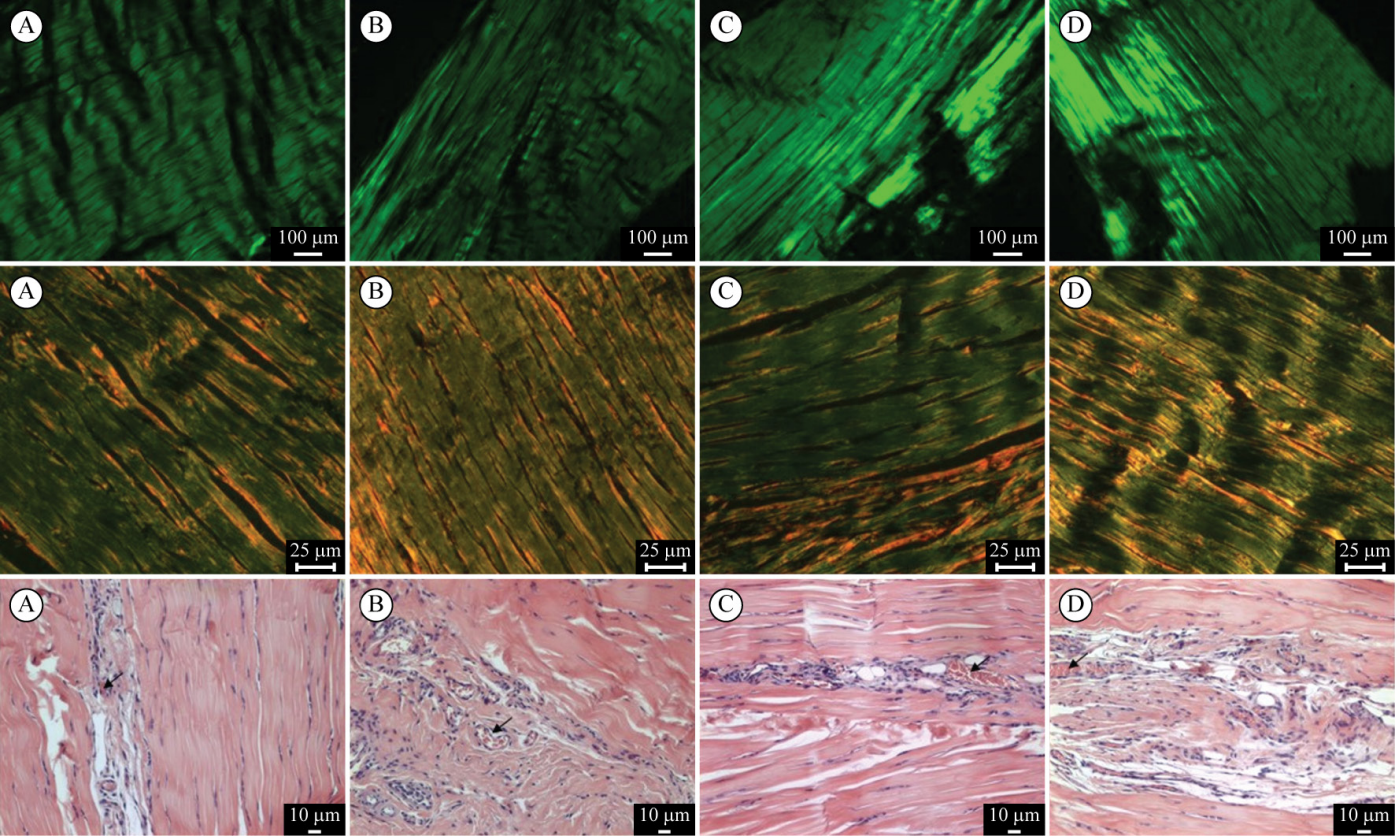
Slides of the Achilles tendon stained with birefringence (first row), picrosirius red (second row), and hematoxylin and eosin (HE) (third row). The letters mean (A) sham; (B) cathodic stimulation; (C) anodic stimulation; and (D) alternating stimulation. The black arrow in the HE slides shows the blood vessels (10x magnification).

No significant differences in optical retardation (F=0.07; p=0.91), percentage of type I (F=0.77; p=0.57) and III (F=0.76; p=0.57) collagen fibers, and numbers of blood vessels (F=0.85; p=0.45) were found between the groups. The data of birefringence measurements, type I collagen fibers and the number of blood vessels showed that the greater the numerical value of the optical retardation, the better the outcome of the HVPC application (Table 1). Figure 2 shows illustrative images of birefringence slides, picrosirius red stained slides, and hematoxylin and eosin stained slides.

**Table 1:** Mean values, standard deviation, inferential analyses, and 95% confidence interval (between parentheses) of optical retardation (OR), types I and III collagen fiber percentage, and blood vessel quantification in the study groups.

	OR (nm)	Collagen fibers (%)		Blood vessel/mm^2^
		Type I	Type III	
Sham	68.02±18.09	14.4±7.1	85.5±7.1	38.17±6.53
	(55.07-80.96)	(9.32-19.47)	(80.42-90.57)	(33.49-42.84)
				
Cathodic	63.33±32.02	15.3±7.8	84.7±7.8	32.26±9.95
	(40.42-86.23)	(9.72-20.87)	(79.12-90.27)	(25.14-39.37)
				
Anodic	66.81±30.11	19.2±10.4	80.8±10.4	38.98±13.59
	(45.27-88.34)	(11.76-26.63)	(73.36-88.23)	(29.25-48.70)
				
Alternating	68.18±23.47	19.4±12.9	80.8±13.0	39.74±13.69
	(51.39-84.96)	(10.17-28.62)	(71.50-90.09)	(29.94-49.53)
				
*p* value	*p*=0.91	*p*=0.57	*p*=0.57	*p*=0.45
F value	0.07	0.77	0.76	0.85

## Discussion

The present study was one of the first to assess the effects of high voltage pulsed current (HVPC) in the healing of partial ruptures of the Achilles tendon in rats by means of birefringence measurements, type I and III collagen fiber percentages, and blood vessel quantification. Our results show that HVPC did not favor collagen realignment or type I collagen fiber increase or number of blood vessels increase.

HVPC has been used in peripheral neurological deficits and vascular and chronic pressure cutaneous ulcer treatments because it speeds up the healing process represented by smaller, better-looking lesions^14^ ^-^ ^18^. Jeon et al.^19^ observed that HVPC with visible contraction intensity was effective in promoting wound healing by increasing expression of transforming growth factor β1(TGF-β1) and synthesis of type I collagen. Furthermore, HVPC has been an effective method for minimizing the severity of repetitive stress injuries of the wrist (pain and edema levels decreased and grip strength and the range of motion increased)^20^.

HVPC was applied on six consecutive days for 30 minutes and the frequency used was 120 pps. This frequency was chosen because some studies have found interesting results with high frequency (100-120 pps): it produced antimicrobial effects in an in vitro study^5^, it reduced edema formation after impact injuries in animals^21^ and it reduced microvessel leakiness^22^. Most studies have used 30 minutes of HVPC with significant results related to tissue healing, such as reduction of edema formation^21^, an effective method for minimizing the severity of repetitive stress injuries^20^. In our study, HVPC was used for six days because our research group found important results related to neovascularization^10^, collagen birefringence^6^ ^,^ ^8^, and the amount of collagen type I and III^8^ ^,^ ^11^ in the first week after tendon injury.

Only Owoeye et al.^7^ conducted a study in rats and observed that HVPC with cathodic polarity increased tendon strength against a new rupture after tenotomy in Achilles tendons. For this reason, the HVPC has probably encouraged the realignment or increased the type I collagen fibers, which is an essential factor for the tendon to acquire resistance to traction forces.

Even though this article did not show the efficacy of HVPC in tendon healing with the analyzed parameters, it is important to highlight that it is one of the studies in the literature that used HVPC for tendon healing, and it is important that more studies be conducted with different parameters: other current frequency; other evaluation periods such as three, 14, or 21 days; or even a study on another lesion model, such as tenotomy followed or not by sutures.

In the findings of the present study, the HVPC did not favor collagen realignment or type I collagen fiber increase, but it is important to observe that the present study’s methodology differed in some aspects, such as partial rupture of the Achilles tendon and the rubber silicon electrodes, instead of the tenotomy followed by sutures and implanted electrodes used by Owoeye et al.^7^. The rubber silicon electrodes were employed because our purpose was to replicate as much as possible the clinical environment, even knowing that it is not possible to correlate it directly. Regarding selected parameters, the present study used 120 pps frequency, while Owoeye et al.^7^ used 10 pps. Therefore, the parameters such as high-frequency electrical current or six days of application can justify the absence of results. Nonetheless, more studies are necessary to investigate the effects on tendon tissue. Owoeye et al.^7^ applied the HVPC for 15 minutes for 14 consecutive days, while the present study opted for 30 minutes for six consecutive days.

The time and frequency of HVPC application are important factors in the healing process. Other studies were found in the literature that obtained satisfactory results with an electrical current for tendon healing, but the evaluations were made 14 days after the injury^23^ ^,^ ^24^. However, there are studies that used low level laser therapy (LLLT) on tendons submitted to partial rupture of the Achilles tendon in rats using the same lesion model as this study, and they observed an increase in collagen realignment^8^ ^,^ ^9^ ^,^ ^11^ and type I collagen percentage after 5 days of treatment^8^ ^,^ ^11^ and increase of blood vessel numbers after 3 and 5 treatment days^10^.

We studied both types of polarity, including alternating polarity, because little is known about the polar effect of this current. A systematic review on the influence of HPVC on edema formation after acute injury in experimental studies observed that a negative polarity is more effective in edema formation management^21^. In view of that observation, this study emphasized other aspects of healing and did not analyze the edema formation observed in the limb submitted to direct trauma. The action mechanism may be related to lower microvessel permeability to plasmatic proteins as observed by Reed^22^, who used negative polarity, and Taylor et al.^25^, who reported satisfactory results with both polarities. Negative polarity^18^ and alternating polarity^15^ ^,^ ^17^ showed satisfactory results in chronic ulcer healing. In this study, the type of polarity did not alter the results. For this reason, it is important that more studies investigate the polar effects.

Some researchers have evaluated the realignment of collagens fibers by birefringence measurements in Achilles tendons. They found significant results after applying pulsed ultrasound (PUS) after a tenotomy^6^ and after direct trauma^11^. Other studies have shown a better collagen alignment through birefringence measurements after acute trauma on tendons treated with LLLT^8^ ^,^ ^9^.

Wood et al.^11^ verified that PUS, LLLT, and the combined use of LLLT and PUS resulted in a greater synthesis of type I collagen in Achilles tendons submitted to direct trauma after five consecutive days, as evaluated by Picrosirius Red. The findings of the present study did not show that HVPC is favorable to collagen fiber realignment or increasing of synthesis after direct trauma to tendons. However, it is important to observe that the mechanisms of tissue interaction are different between the physical agents. Due to the lack of studies on HVPC in tendon healing, it is important that studies with other parameters of this electrical current be carried out.

Given the importance of blood vessel formation for oxygen and nutrient supply that help in the healing process, the blood vessels were counted in the tendon central area, which is poor in vascularization. However, it is relevant to mention that in future studies, blood vessels should also be counted in the peritendinous sheath. The literature shows that HVPC did not promote angiogenesis in the neural tissue^26^, and in this study, we could see the same results. However, HVPC increased the blood flow speed^27^ and increased microcirculation in the skin wounds^28^. Furthermore, increased capillary numbers were observed on day 14 after applying rectangular, biphasic, symmetric, and pulsed electrical currents to tenotomized and sutured Achilles tendons in rats^23^.

There are other important results using different types of current for electrical stimulation, such as enhancement of intrinsic tenoblastic repair in vitro^29^ and increase in capillaries and fibroblasts at the early stage of tissue repair^23^; in terms of low voltage microamperage stimulation, an improved tensile strength of partially transected Achilles tendons at four weeks after injury^24^ and burst transcutaneous electrical nerve stimulation (TENS) that inhibited collagen I and III production and impaired its alignment during healing of partial rupture of the Achilles tendon in rats^30^. More studies should be conducted with different parameters, e.g., analysis of collagen synthesis using real-time polymerase chain reaction (RT-PCR) or immunohistochemistry, identifying some key genes and proteins important for healing tendon such as vascular endothelial growth factor (VEGF), transforming growth factor-β (TGF-β), insulin-like growth factor (IGF), connective tissue growth factor (CTGF), matrix metalloproteinases (MMPs), tenascin C, and important proteoglycans.

## Conclusions

In conclusion, daily 30-minute applications of HVPC on six consecutive days were not effective in collagen realignment and synthesis and in angiogenesis after partial Achilles tendon rupture in rats. Therefore, these findings do not support the use of HVPC to improve tendon repair; however, they should be confirmed by clinical trials.
